# Amphetamine-Associated Reversible Biventricular Cardiomyopathy Complicated by Acute Respiratory Failure: A Case Report

**DOI:** 10.7759/cureus.114490

**Published:** 2026-08-13

**Authors:** Bijay Parajuli, Sajja Sharma, Yogesh Subedi, Milan Guragain, Binita Bhandari

**Affiliations:** 1 Internal Medicine, University of Pittsburgh Medical Center (UPMC) Health System, Cumberland, USA; 2 Internal Medicine, Nepalese Army Institute of Health Sciences, Kathmandu, NPL; 3 Hospital Medicine, University of Pittsburgh Medical Center (UPMC) Health System, Cumberland, USA; 4 Cardiology, West Virginia University, Morgantown, USA

**Keywords:** adderall overdose, amphetamine-induced cardiomyopathy, ards, biventricular cardiomyopathy, reversible cardiomyopathy, stimulant cardiotoxicity

## Abstract

Amphetamine overdose can cause severe cardiovascular toxicity, including rarely reversible cardiomyopathy. A 26-year-old man presented approximately 33 hours after intentionally ingesting 25 20-mg extended-release mixed amphetamine salt tablets with lorazepam. He developed chest pain, tachycardia, hypotension, hypoxemia, lactic acidosis, rhabdomyolysis, and markedly elevated cardiac biomarkers. Electrocardiography showed diffuse ST-segment depressions, and chest imaging demonstrated bilateral pulmonary edema and acute lung injury without pulmonary embolism. Echocardiography revealed severe global biventricular systolic dysfunction with a left ventricular ejection fraction below 20%. Progressive hypoxemic respiratory failure required intubation and mechanical ventilation. He improved with supportive intensive care, intravenous diuresis, and heart failure therapy. Although coronary angiography was recommended, he declined and left against medical advice. At two-month follow-up, echocardiography showed complete recovery of biventricular function, with an ejection fraction of 55-60%. This case highlights profound but reversible biventricular cardiomyopathy following massive extended-release amphetamine overdose.

## Introduction

Amphetamine-based stimulants, including mixed amphetamine salts, are commonly prescribed for attention-deficit/hyperactivity disorder but are also frequently misused. Although their cardiovascular effects are often limited to tachycardia, hypertension, and palpitations, severe complications such as coronary vasospasm, arrhythmias, myocardial ischemia, stress cardiomyopathy, and dilated cardiomyopathy have been reported [1,2]. The mechanism is thought to involve excessive catecholamine stimulation, increased myocardial oxygen demand, coronary microvascular spasm, oxidative stress, and direct myocardial toxicity [3-5].

Most published reports of amphetamine-associated cardiomyopathy describe chronic methamphetamine exposure or Takotsubo-like cardiomyopathy following amphetamine/dextroamphetamine ingestion [4,6,7]. Severe acute biventricular cardiomyopathy after massive extended-release mixed amphetamine salt ingestion, particularly when complicated by hypoxemic respiratory failure requiring mechanical ventilation, is less commonly described. Importantly, myocardial dysfunction may be reversible when the injury is recognized early and managed supportively [2].

We report a case of a 26-year-old male who developed severe reversible biventricular cardiomyopathy and acute hypoxemic respiratory failure after intentional ingestion of approximately 500 mg of extended-release mixed amphetamine salts. Despite the initial left ventricular ejection fraction of less than 20% and right ventricular dysfunction, follow-up echocardiography two months later demonstrated complete recovery of biventricular systolic function.

## Case presentation

A 26-year-old male with a past medical history significant for depression presented to the emergency department via emergency medical services approximately 33 hours after intentionally ingesting approximately 25 20-mg extended-release mixed amphetamine salt tablets (Adderall XR), corresponding to a total estimated dose of 500 mg. The patient also reported ingesting five 0.5 mg lorazepam tablets. He stated that he had been experiencing worsening depressive symptoms and suicidal ideation and purchased Adderall tablets illicitly because he believed they would improve his mood. Before presentation, he experienced progressive dizziness, lightheadedness, chest pain, and shortness of breath.

Emergency medical services administered 324 mg aspirin, 4 mg midazolam, and intravenous fluids during transport. Upon arrival to the emergency department, the patient was afebrile but appeared acutely ill and lethargic. Initial vital signs demonstrated a heart rate of 158 beats/minute, a respiratory rate of 37 breaths/minute, blood pressure of 98/80 mmHg, and oxygen saturation of 88% on room air. Due to worsening hypoxemia, bilevel positive airway pressure was initiated, with improvement of oxygen saturation to 98%.

Physical examination revealed a lethargic but arousable young male. Cardiac examination demonstrated marked tachycardia without audible murmurs. Pulmonary examination revealed respiratory distress with diffuse crackles bilaterally. No peripheral edema was present. Neurologic examination was nonfocal.

Laboratory evaluation demonstrated marked leukocytosis (24.2 × 10³/µL), elevated high-sensitivity troponin I (11,407 ng/L), elevated B-type natriuretic peptide (BNP) (2,155 pg/mL), elevated creatine kinase (1,185 U/L), elevated lactate (4.6 mmol/L), mild transaminitis, and metabolic abnormalities including hyponatremia and hyperkalemia (Table 1). Urine toxicology was positive for amphetamines, benzodiazepines, methadone, and tetrahydrocannabinol. Screening for COVID-19, influenza A/B, and respiratory syncytial virus was negative. Respiratory cultures remained negative throughout hospitalization (Table 2).

**Table 1:** Laboratory investigations. WBC = white blood cell count; BNP = B-type natriuretic peptide; CK = creatine kinase; AST = aspartate aminotransferase; ALT = alanine aminotransferase

Parameter	Admission	Peak/Lowest	Discharge	Reference range
WBC (×10³/μL)	24.2	24.2	7.2	4.0–10.8
Troponin I (ng/L)	11,407	11,407/2510	—	<19
BNP (pg/mL)	2,155	3,063	—	<100
CK (U/L)	1,185	1,185	212	49–275
Lactate (mmol/L)	4.6	4.6	0.9	0.5–2.5
Sodium (mmol/L)	133	129	134	136–144
Potassium (mmol/L)	5.5	3.1	3.1	3.5–5.1
AST (U/L)	89	89	38	15–41
ALT (U/L)	42	54	54	10–54
Creatinine (mg/dL)	1.1	1.5	0.9	0.7–1.2

**Table 2:** Microbiological evaluation. PCR = polymerase chain reaction; RSV = respiratory syncytial virus; MRSA = methicillin-resistant *Staphylococcus aureus*

Test	Result
COVID-19 PCR	Negative
Influenza A	Negative
Influenza B	Negative
RSV PCR	Negative
MRSA PCR	Negative
Sputum Gram stain	No organisms seen
Respiratory culture	No growth after 48 hours

**Figure 1:**
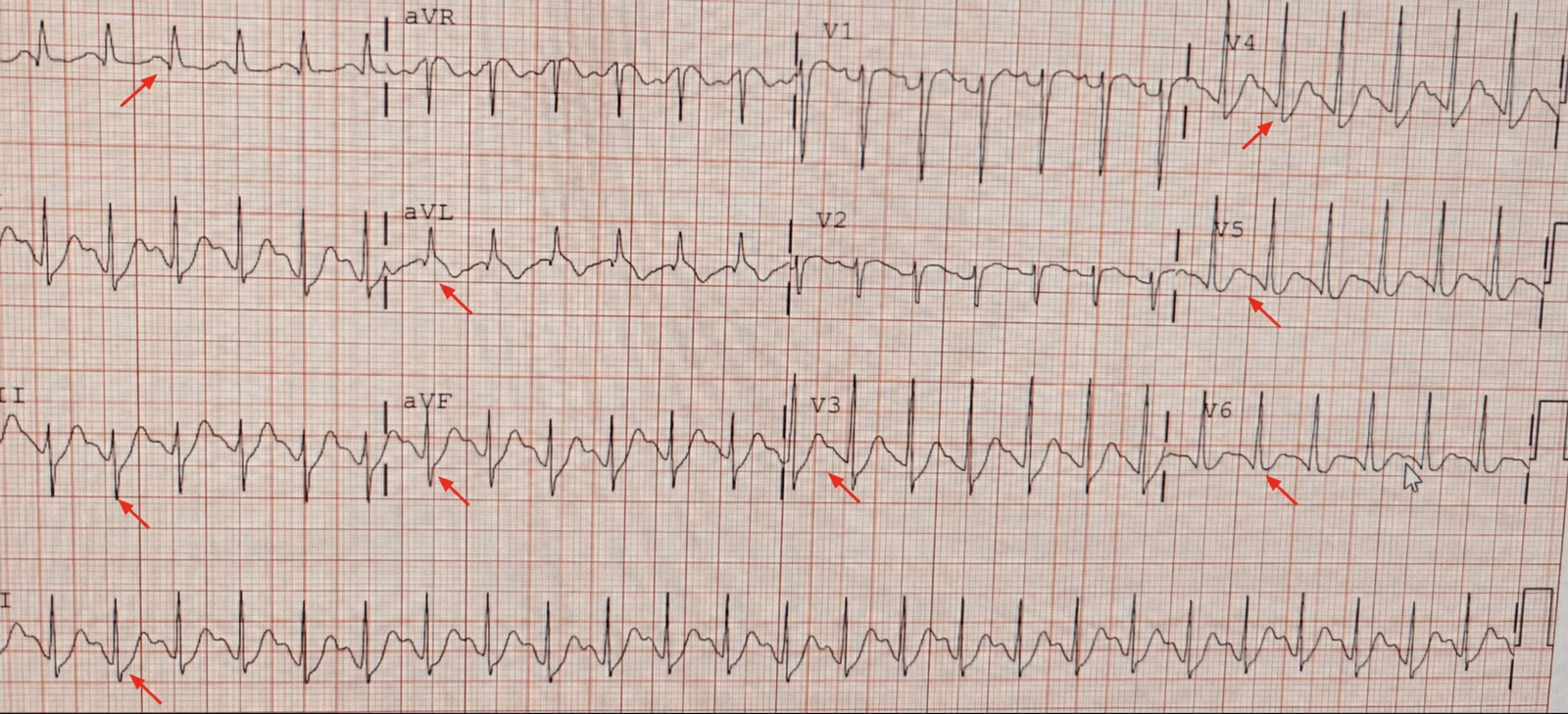
Admission 12-lead electrocardiogram demonstrating sinus tachycardia with diffuse ST-segment depressions involving the inferior and anterolateral leads, without ST elevation.

Electrocardiography demonstrated sinus tachycardia with a ventricular rate of 155 beats/minute, corrected QT interval of 523 ms, and diffuse ST-segment depressions involving the inferior and anterolateral leads without ST-segment elevation (Figure 1). Point-of-care ultrasound demonstrated diffuse bilateral B-lines consistent with pulmonary edema.

**Figure 2:**
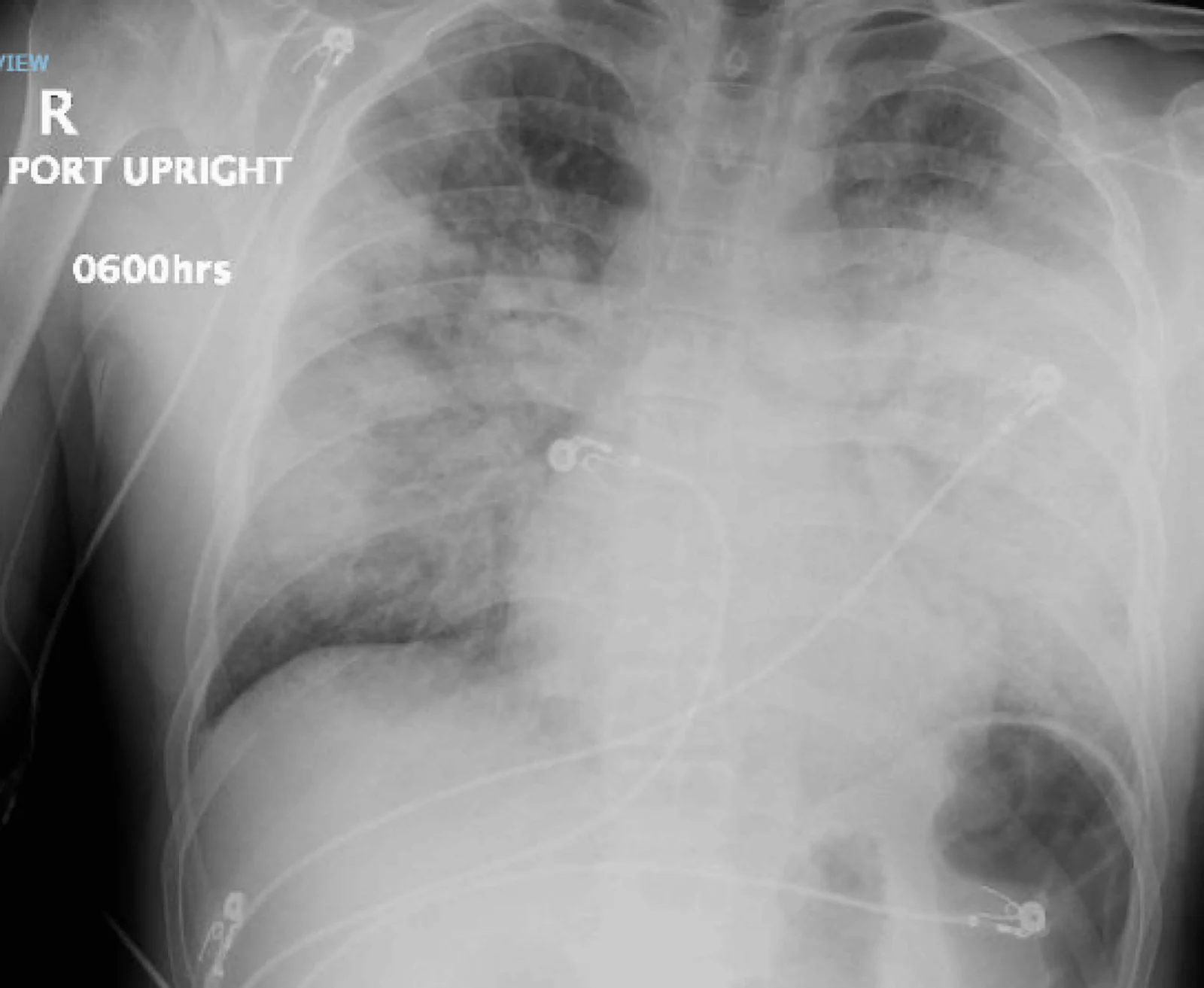
Admission chest radiograph. Portable chest radiograph obtained on admission demonstrating diffuse bilateral patchy air-space opacities, most prominent in the perihilar and upper lung fields, consistent with acute pulmonary edema and acute lung injury. Findings correlate with the patient’s severe hypoxemic respiratory failure and acute cardiomyopathy.

**Figure 3:**
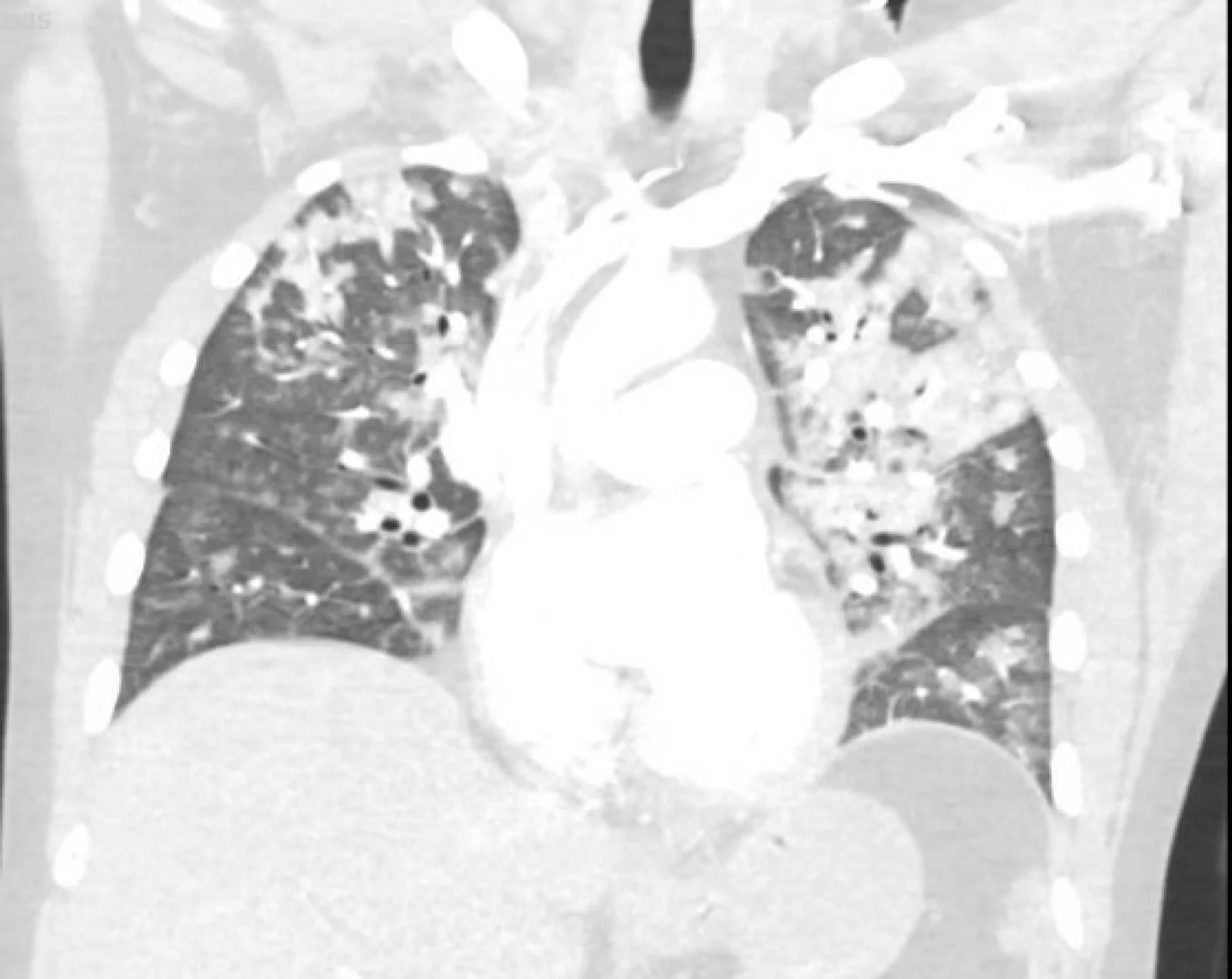
Computed tomography angiography of the chest. Coronal computed tomography angiography of the chest demonstrating bilateral multifocal ground-glass and consolidative pulmonary opacities involving both lungs, with greater involvement of the upper lobes. No evidence of pulmonary embolism was identified. Findings were consistent with pulmonary edema and acute lung injury in the setting of severe amphetamine-induced cardiomyopathy.

Admission chest radiography revealed extensive bilateral air-space opacities consistent with pulmonary edema (Figure 2). Repeat chest radiography following respiratory deterioration demonstrated persistent diffuse bilateral infiltrates requiring endotracheal intubation. Computed tomography angiography of the chest excluded pulmonary embolism but demonstrated bilateral multifocal ground-glass and consolidative pulmonary infiltrates with findings suggestive of pulmonary edema and acute lung injury (Figure 3).

**Figure 4:**
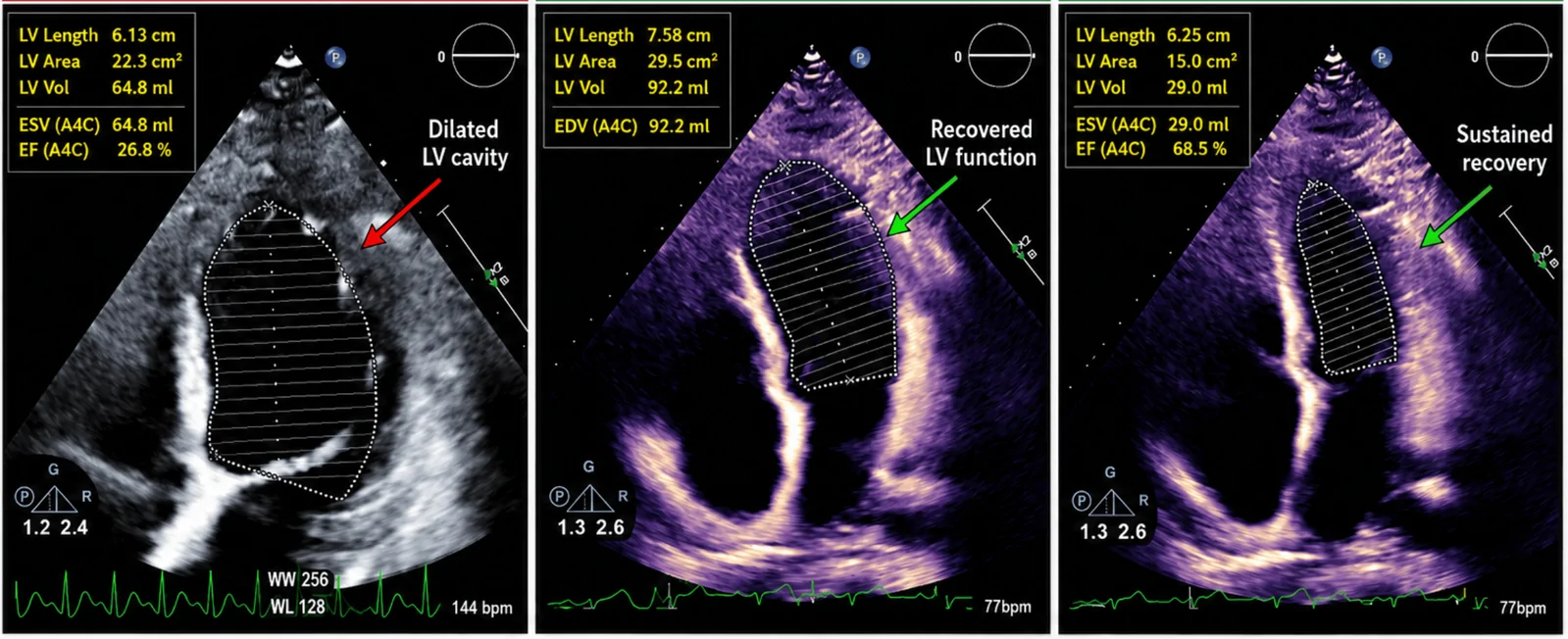
Transthoracic echocardiography demonstrating recovery of left ventricular systolic function. Apical four-chamber transthoracic echocardiographic views with left ventricular tracing. The initial study demonstrated markedly reduced left ventricular systolic function in the setting of acute amphetamine-associated cardiomyopathy, while follow-up imaging showed normalization of left ventricular ejection fraction to 68.5%, supporting reversible stress/toxin-mediated cardiomyopathy.

Urgent transthoracic echocardiography revealed severe global left ventricular systolic dysfunction with estimated left ventricular ejection fraction of less than 20%, severe global hypokinesis, and concomitant right ventricular dysfunction. No significant valvular abnormalities were identified (Table 3, Figure 4). Given the marked troponin elevation and severe ventricular dysfunction, ischemic evaluation was recommended by the cardiology service.

**Table 3:** Echocardiographic findings at presentation and follow-up.

Echocardiographic parameter	Initial echocardiogram	Follow-up echocardiogram
Left ventricular ejection fraction	Less than 20%; apical four-chamber calculated ejection factor of 26.8%	55–60%; apical four-chamber calculated ejection factor of 68.5%
Left ventricular systolic function	Severely reduced	Normalized
Left ventricular wall motion	Severe global hypokinesis	Normal wall motion
Right ventricular function	Reduced	Normalized
Left ventricular end-diastolic volume	88.5 mL	92.2 mL
Left ventricular end-systolic volume	64.8 mL	29.0 mL
Clinical interpretation	Acute severe biventricular cardiomyopathy	Complete recovery of biventricular systolic function

Despite initial noninvasive ventilatory support, the patient developed worsening acute hypoxemic respiratory failure and required endotracheal intubation and mechanical ventilation. Intensive care management included ventilatory support, intravenous diuresis with furosemide, electrolyte optimization, and guideline-directed heart failure therapy as tolerated. Empiric antibiotic therapy was initiated for possible superimposed pneumonia; however, microbiologic studies remained negative.

The patient’s respiratory status gradually improved, allowing successful extubation on hospital day four. Serial laboratory studies demonstrated normalization of lactate levels, improvement in transaminases, resolution of leukocytosis, and decreasing cardiac biomarkers. Repeat inpatient echocardiography continued to demonstrate significant left ventricular dysfunction. Coronary angiography was recommended to exclude obstructive coronary disease; however, the patient declined invasive evaluation and subsequently left the hospital against medical advice. The patient later returned for outpatient cardiology follow-up. Repeat transthoracic echocardiography approximately two months after discharge demonstrated complete recovery of left and right ventricular systolic function with left ventricular ejection fraction between 55% and 60% (Table 3, Figure 4). The dramatic improvement in ventricular function strongly supported a diagnosis of reversible amphetamine-induced cardiomyopathy.

**Figure 5:**
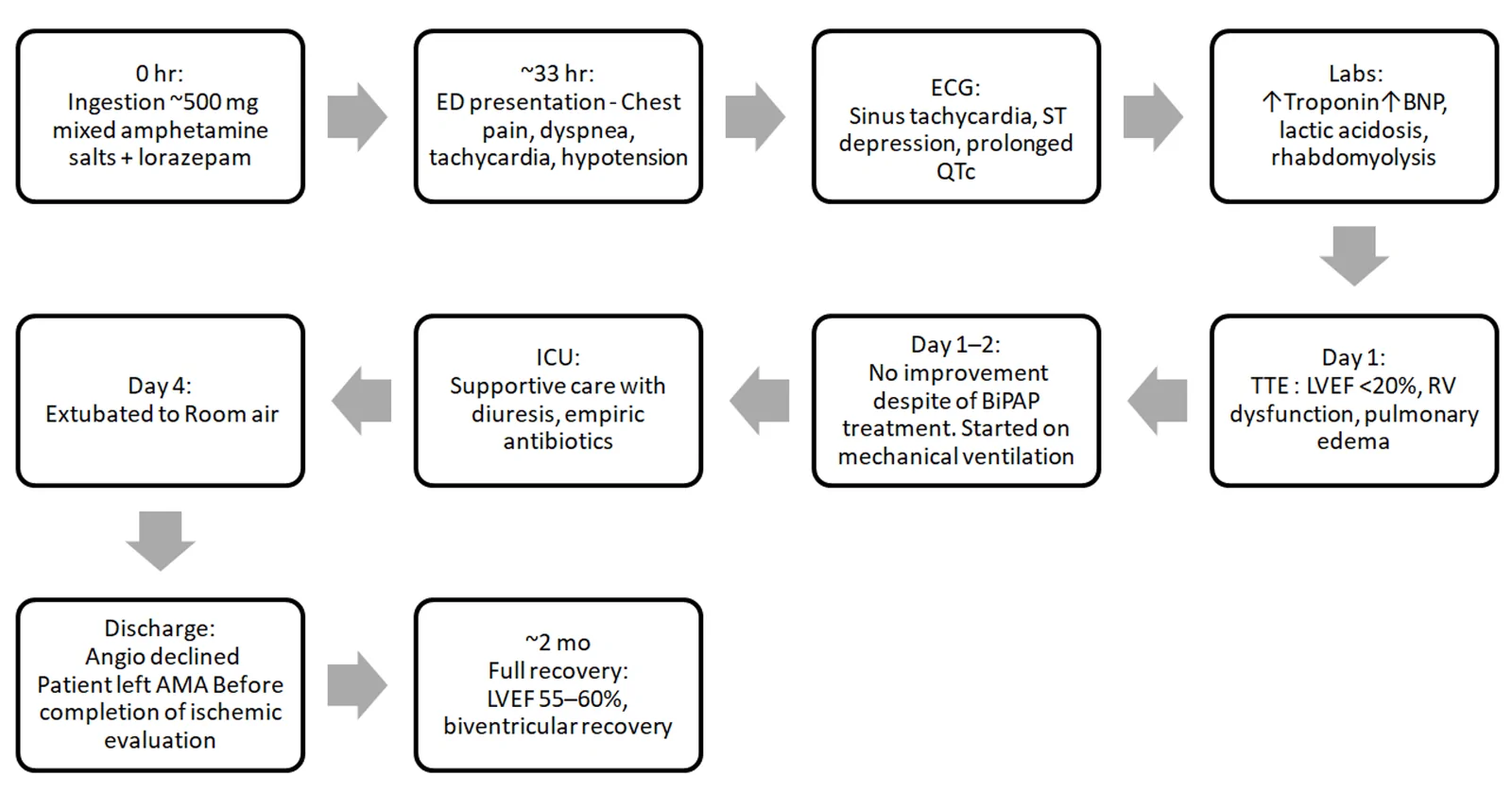
Clinical timeline of amphetamine-induced cardiopulmonary toxicity and recovery. The timeline depicts the reported ingestion, delayed presentation, development of severe biventricular cardiomyopathy and hypoxemic respiratory failure, intensive care treatment, extubation, discharge against medical advice, and complete biventricular recovery at approximately two months. ED = emergency department; ECG = electrocardiography; BNP = B-type natriuretic peptide; BiPAP = bilevel positive airway pressure; LEVF = left ventricular ejection fraction; AMA = against medical advice

## Discussion

Amphetamine-associated cardiomyopathy is an increasingly recognized complication of stimulant exposure, particularly with misuse, supratherapeutic dosing, or overdose [1-4]. Amphetamines increase synaptic catecholamine concentrations, producing sustained sympathetic stimulation, tachycardia, coronary vasoconstriction, increased myocardial oxygen demand, oxidative stress, and direct myocardial toxicity [1-5]. These mechanisms can result in myocardial ischemia, myocyte injury, stress cardiomyopathy, or dilated cardiomyopathy [1-5]. Although most literature has focused on chronic methamphetamine use, acute high-dose exposure to mixed amphetamine salts can also produce severe but potentially reversible myocardial dysfunction [4,6,7].

The presentation was notable for chest pain, marked sinus tachycardia, diffuse ST-segment depressions, prolonged corrected QT interval, severe troponin elevation, elevated BNP, pulmonary edema, and acute hypoxemic respiratory failure requiring mechanical ventilation. Initial transthoracic echocardiography demonstrated severe global left ventricular hypokinesis with left ventricular ejection fraction of less than 20% and associated right ventricular dysfunction. The complete recovery of biventricular function at approximately two months strongly supports a reversible toxic cardiomyopathy rather than fixed structural heart disease.

The pathophysiology in this case was likely multifactorial. Excess catecholamine activity from amphetamine toxicity can cause coronary vasospasm, calcium overload, mitochondrial dysfunction, oxidative injury, and direct myocyte necrosis [2-5]. Histopathologic studies of methamphetamine-associated cardiomyopathy have described contraction band necrosis, myocytolysis, and interstitial fibrosis, supporting a catecholamine-mediated injury pattern [5]. However, in acute toxicity, myocardial dysfunction may reflect transient myocardial stunning rather than irreversible fibrosis. This distinction is clinically important because recovery is possible when exposure stops and supportive care is provided [4,8-11]. The rapid normalization of systolic function in our patient is consistent with reversible myocardial stunning due to acute stimulant toxicity.

The differential diagnosis included acute coronary syndrome, coronary vasospasm, stress-induced cardiomyopathy, myocarditis, sepsis-related cardiomyopathy, and toxic cardiomyopathy (Table 4). Acute coronary syndrome was considered because of chest pain, diffuse ST-segment depressions, and marked troponin elevation. Coronary angiography was recommended but declined by the patient, who left the hospital against medical advice; therefore, obstructive coronary disease could not be definitively excluded. However, the patient’s young age, massive stimulant exposure, global rather than regional ventricular dysfunction, positive amphetamine screen, and complete recovery of left and right ventricular function favored amphetamine-induced reversible cardiomyopathy. Myocarditis was also considered but was less likely given the absence of a viral prodrome, negative viral testing, strong temporal relationship with overdose, and recovery without myocarditis-specific therapy.

**Table 4:** Differential diagnosis of acute severe cardiomyopathy (possible for this patient).

Diagnosis	Supporting features	Features arguing against or limiting diagnosis
Amphetamine-induced cardiomyopathy	Massive reported amphetamine ingestion, positive amphetamine screen, severe tachycardia, global biventricular dysfunction, pulmonary edema, complete recovery	Illicitly obtained tablets create the possibility of unrecognized co-ingestants. Coronary angiogram, cardiac magnetic resonance imaging, epicardial biopsy, and full viral panel were not done, which makes amphetamine use a possible cause and not definite
Acute coronary syndrome	Chest pain, markedly elevated troponin, diffuse ST-segment depressions	Young age, no ST-segment elevation, global rather than regional dysfunction
Coronary vasospasm	Stimulant exposure, chest pain, ST-segment changes, troponin elevation	Not directly proven because coronary angiography was not performed
Takotsubo cardiomyopathy	Catecholamine surge, reversible systolic dysfunction	Global biventricular dysfunction rather than classic regional Takotsubo pattern
Myocarditis	Troponin elevation, severe systolic dysfunction	No viral prodrome, negative viral testing, strong toxic exposure history; no cardiac magnetic resonance imaging or biopsy performed
Sepsis-related cardiomyopathy	Leukocytosis and pulmonary infiltrates	Negative microbiologic testing, no confirmed infectious source, improvement with diuresis and supportive care

This case also highlights the pulmonary toxicity associated with severe amphetamine-induced cardiomyopathy. The patient developed acute hypoxemic respiratory failure with diffuse bilateral pulmonary infiltrates on chest radiography and multifocal ground-glass and consolidative opacities on computed tomography angiography. These findings were consistent with pulmonary edema and acute lung injury with acute respiratory distress syndrome-like physiology. The markedly elevated BNP, diffuse B-lines on point-of-care ultrasound, severe left ventricular dysfunction, and clinical improvement with intravenous diuresis supported a major cardiogenic pulmonary edema component. Infectious causes were considered; however, respiratory viral testing was negative, sputum Gram stain showed no organisms, and lower respiratory culture demonstrated no growth. Therefore, the pulmonary findings were most consistent with cardiogenic pulmonary edema and toxic acute lung injury rather than primary infectious pneumonia.

Several prior reports have described reversible cardiomyopathy after amphetamine or amphetamine/dextroamphetamine exposure. Toce et al. reported reversible Takotsubo cardiomyopathy after amphetamine/dextroamphetamine ingestion in an adolescent [6], and Alsidawi et al. described Adderall-induced inverted Takotsubo cardiomyopathy [7]. Other reports and reviews have described amphetamine-associated dilated cardiomyopathy and variable recovery depending on ongoing exposure and degree of myocardial fibrosis [2,8,9,11-13]. Compared with these reports, our case is notable for severe biventricular dysfunction after massive extended-release Adderall ingestion, acute hypoxemic respiratory failure requiring intubation, and complete recovery of systolic function within two months (Table 5).

**Table 5:** Comparison with selected published reports of amphetamine-associated reversible cardiomyopathy.

Study	Patient population/Case	Exposure	Cardiac phenotype	Recovery
Toce et al. [6]	15-year-old adolescent	Amphetamine/Dextroamphetamine ingestion	Reversible Takotsubo cardiomyopathy	Complete recovery
Alsidawi et al. [7]	Adult patient	Adderall	Inverted Takotsubo cardiomyopathy	Complete recovery
Ayres [12]	Case report	Amphetamine	Amphetamine-associated cardiomyopathy	Noted association
O’Hara [13]	Case report/Commentary	Amphetamines	Dilated nonischemic cardiomyopathy with atrial fibrillation	Variable clinical course
Present case	26-year-old male	Massive reported Adderall XR ingestion	Severe reversible biventricular cardiomyopathy with acute hypoxemic respiratory failure requiring mechanical ventilation	Complete biventricular recovery at approximately two months

Management of amphetamine-associated cardiomyopathy is primarily supportive [2,4,11]. Initial care should focus on airway and oxygenation support, treatment of agitation, correction of electrolyte abnormalities, arrhythmia monitoring, and management of acute heart failure [2,4]. In patients with pulmonary edema and adequate perfusion, cautious intravenous diuresis is appropriate. Guideline-directed medical therapy for heart failure with reduced ejection fraction should be started as tolerated, although the optimal duration of therapy after complete recovery remains uncertain [4,11]. Psychiatric evaluation and substance-use counseling should be incorporated into management, particularly following an intentional overdose, to address underlying psychiatric conditions and reduce the risk of recurrent stimulant exposure [2,4].

This case has important limitations. Coronary angiography was not performed because the patient declined invasive ischemic evaluation and left against medical advice. Cardiac magnetic resonance imaging and endomyocardial biopsy were also not performed, limiting definitive exclusion of myocarditis or myocardial fibrosis. This is relevant because prior studies suggest that myocardial fibrosis, particularly when identified by late gadolinium enhancement on cardiac magnetic resonance imaging, may influence the likelihood of recovery in stimulant-associated cardiomyopathy [8,9]. In addition, the reported Adderall tablets were purchased illicitly, raising the possibility of unrecognized co-ingestants or counterfeit substances. Despite these limitations, the temporal association with massive stimulant ingestion, positive amphetamine screen, acute global biventricular dysfunction, cardiopulmonary improvement with supportive care, and complete recovery on follow-up support amphetamine-induced reversible biventricular cardiomyopathy.

Clinicians should maintain a high index of suspicion for stimulant-induced cardiomyopathy in young patients presenting with chest pain, tachycardia, elevated cardiac biomarkers, pulmonary edema, and new severe ventricular dysfunction [1-4,10,11]. Early echocardiography, careful supportive management, and close follow-up are essential because even profound systolic dysfunction may be reversible when stimulant exposure is discontinued and appropriate supportive care is provided [4,8-11].

## Conclusions

This case demonstrates an unusual presentation of profound, reversible biventricular cardiomyopathy following a massive extended-release mixed amphetamine salt overdose. Distinctive features include presentation approximately 33 hours after ingestion, severe global dysfunction of both ventricles, concurrent pulmonary edema and acute hypoxemic respiratory failure requiring mechanical ventilation, and complete recovery of biventricular systolic function within two months. Although obstructive coronary disease could not be definitively excluded because coronary angiography was declined, the temporal relationship to overdose, positive amphetamine screen, global rather than regional ventricular dysfunction, and subsequent normalization of cardiac function supported possible stimulant-induced cardiomyopathy. This case helps to add more information to the limited literature on acute biventricular failure after prescription amphetamine overdose and demonstrates that complete myocardial recovery may occur despite delayed presentation and life-threatening cardiopulmonary dysfunction. Early echocardiography, supportive intensive care, careful diuresis, electrolyte optimization, heart failure therapy as tolerated, psychiatric evaluation, avoidance of further stimulant exposure, and close follow-up remain important.

## Appendices

Appendix 1

The positive amphetamine screen supported the reported ingestion, while other findings were considered when evaluating possible contributors to cardiopulmonary toxicity.

**Table 6:** Urine toxicology.

Test	Result	Clinical relevance
Urine amphetamine screen	Positive	Supports reported stimulant exposure
Urine benzodiazepine screen	Positive	Consistent with reported lorazepam ingestion and emergency medical services-administered midazolam
Urine methadone screen	Positive	Possible additional exposure or toxicology-screen limitation; may contribute to corrected QT interval prolongation
Urine tetrahydrocannabinol screen	Positive	Indicates cannabis exposure
Urine cocaine screen	Negative	Makes cocaine-associated vasospasm less likely
Urine fentanyl screen	Negative	No fentanyl detected
Urine opiate screen	Negative	No opiates detected
Acetaminophen level	Undetectable	No evidence of acetaminophen co-ingestion
Salicylate level	Undetectable	No evidence of salicylate co-ingestion
Serum ethanol	Not detected	No ethanol detected
